# Supplementary material for: Identification of Novel tet(X3) Variants Resistant To Tigecycline in Acinetobacter Species
Source: Microbiol Spectr. 2022 Nov 21;10(6):e01333-22. doi: 10.1128/spectrum.01333-22 (PMC9784759; doi:10.1128/spectrum.01333-22)
Supplement: Supplemental file 1 — Supplemental material. Download spectrum.01333-22-s0001.pdf, PDF file, 0.3 MB [file spectrum.01333-22-s0001.pdf]

Supplementary file

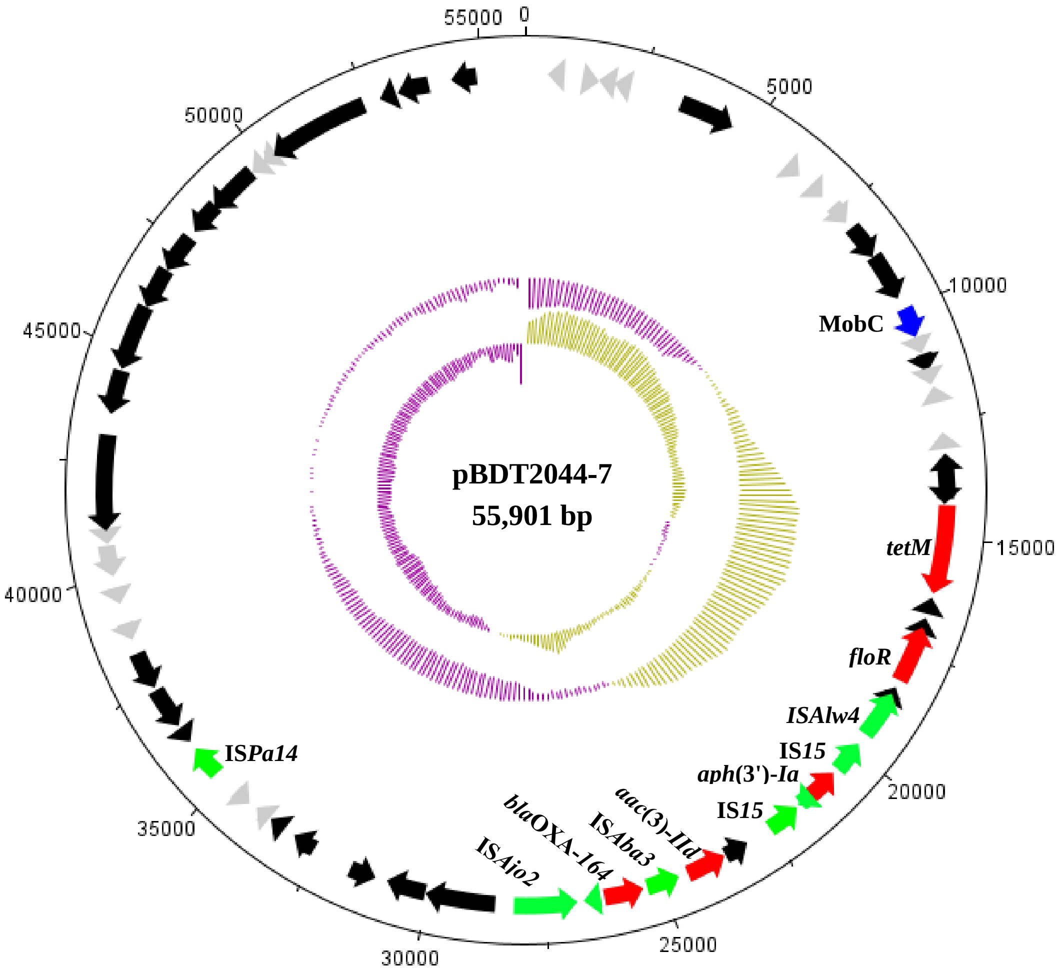

Figure S1. Structure of the index plasmid pBDT2044-7. GC skew and GC content are indicated from the inside out. Positions and transcriptional directions of the predicted ORFs are denoted with arrows. Genes associated with the antimicrobial resistance, mobile element, plasmid mobilization relaxosome protein, other protein and hypothetical protein are highlighted in red, green, blue, black and gray, respectively.

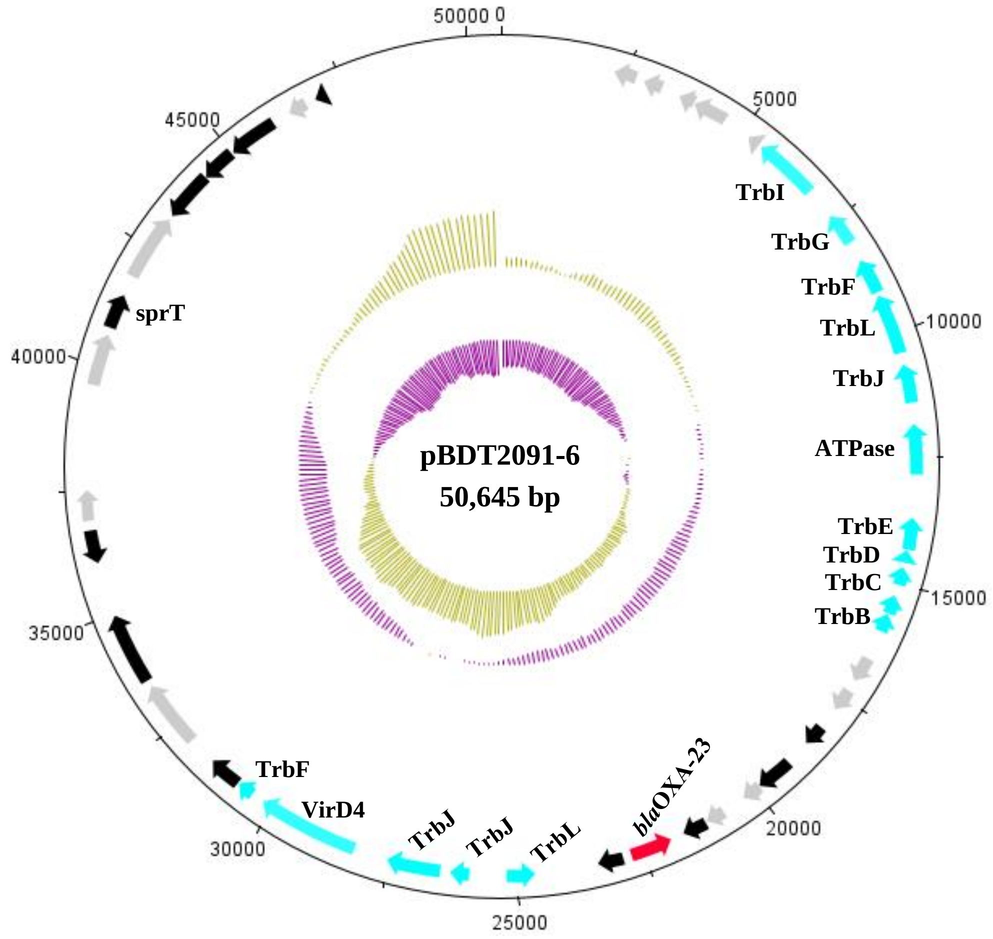

Figure S2. Structure of the conjugative plasmid pBDT2091-6. GC skew and GC content are indicated from the inside out. Positions and transcriptional directions of the predicted ORFs are denoted with arrows. Genes associated with the antimicrobial resistance, conjugative transfer protein, other protein and hypothetical protein are highlighted in red, azure, black and gray, respectively.

Table S1. Primers used in this study.

Primer designation	Sequence (5'-3')	Annealingtem (ºC)	Product size(bp)
tet(X3)-F	ATGACAATGCGAATAGATAC	58.0	1167
tet(X3)-R	TTATAGATTCAATAATTTTTGAAAC		
16S rRNA-F	AGAGTTTGATCCTGGCTCAG	55.0	1400
16S rRNA-R	GGTTACCTTGTTACGACTT		
tet(X3)-inverse-F	CGACAGAAAACGAAACCGAA	57.5	5117
tet(X3)-inverse R	TTCTTGCCTCTCGGTCGTTG		
tet(X3)-EcoRI-F	TACGCGAATTCCGTATAGAACAGTCAAAAGG	58.0	1896
tet(X3)-XbalI-R	TACGCTCTAGAGGCTCCTTTGGGCATACTC		
pro-EcoRI-F	TACGCGAATTCTAAGAACATACTTTATGG	49.0	522
pro-XbalI-R	TACGCTCTAGATCAAAAATGGCACATAACAA		
Gene-SalI-F	TACGCGTCGACATGACAATGCGAATAGATAC	56.0	1189
Gene-HindIII-R	TACGCAAGCTTTTATAGATTCAATAATTTTTGAAAC		
